# The Real-World Experiences of Persons With Multiple Sclerosis During the First COVID-19 Lockdown: Application of Natural Language Processing

**DOI:** 10.2196/37945

**Published:** 2022-11-10

**Authors:** Deborah Chiavi, Christina Haag, Andrew Chan, Christian Philipp Kamm, Chloé Sieber, Mina Stanikić, Stephanie Rodgers, Caroline Pot, Jürg Kesselring, Anke Salmen, Irene Rapold, Pasquale Calabrese, Zina-Mary Manjaly, Claudio Gobbi, Chiara Zecca, Sebastian Walther, Katharina Stegmayer, Robert Hoepner, Milo Puhan, Viktor von Wyl

**Affiliations:** 1 Institute for Implementation Science in Health Care University of Zurich Zurich Switzerland; 2 Epidemiology, Biostatistics and Prevention Institute University of Zurich Zurich Switzerland; 3 Department of Neurology Inselspital, Bern University Hospital and University of Bern Bern Switzerland; 4 Neurocenter Lucerne Cantonal Hospital Lucerne Switzerland; 5 Service of Neurology, Department of Clinical Neurosciences Lausanne University Hospital and University of Lausanne Lausanne Switzerland; 6 Department of Neurology and Neurorehabilitation Rehabilitation Centre Kliniken Valens Valens Switzerland; 7 Division of Molecular and Cognitive Neuroscience University of Basel Basel Switzerland; 8 Department of Neurology Schulthess Klinik Zurich Switzerland; 9 Department of Health Sciences and Technology ETH Zurich Zurich Switzerland; 10 Multiple Sclerosis Center, Department of Neurology Neurocenter of Southern Switzerland Ente Ospedaliero Cantonale Lugano Switzerland; 11 Faculty of Biomedical Sciences Università della Svizzera Italiana (USI) Lugano Switzerland; 12 Translational Research Center University Hospital of Psychiatry and Psychotherapy University of Bern Bern Switzerland

**Keywords:** natural language processing, multiple sclerosis, COVID-19, nervous system disease, nervous system disorder, textual data, health data, patient data, topic modeling, sentiment analysis, linguistic inquiry, medical informatics, clinical informatics

## Abstract

**Background:**

The increasing availability of “real-world” data in the form of written text holds promise for deepening our understanding of societal and health-related challenges. Textual data constitute a rich source of information, allowing the capture of lived experiences through a broad range of different sources of information (eg, content and emotional tone). Interviews are the “gold standard” for gaining qualitative insights into individual experiences and perspectives. However, conducting interviews on a large scale is not always feasible, and standardized quantitative assessment suitable for large-scale application may miss important information. Surveys that include open-text assessments can combine the advantages of both methods and are well suited for the application of natural language processing (NLP) methods. While innovations in NLP have made large-scale text analysis more accessible, the analysis of real-world textual data is still complex and requires several consecutive steps.

**Objective:**

We developed and subsequently examined the utility and scientific value of an NLP pipeline for extracting real-world experiences from textual data to provide guidance for applied researchers.

**Methods:**

We applied the NLP pipeline to large-scale textual data collected by the Swiss Multiple Sclerosis (MS) registry. Such textual data constitute an ideal use case for the study of real-world text data. Specifically, we examined 639 text reports on the experienced impact of the first COVID-19 lockdown from the perspectives of persons with MS. The pipeline has been implemented in Python and complemented by analyses of the “Linguistic Inquiry and Word Count” software. It consists of the following 5 interconnected analysis steps: (1) text preprocessing; (2) sentiment analysis; (3) descriptive text analysis; (4) unsupervised learning–topic modeling; and (5) results interpretation and validation.

**Results:**

A topic modeling analysis identified the following 4 distinct groups based on the topics participants were mainly concerned with: “contacts/communication;” “social environment;” “work;” and “errands/daily routines.” Notably, the sentiment analysis revealed that the “contacts/communication” group was characterized by a pronounced negative emotional tone underlying the text reports. This observed heterogeneity in emotional tonality underlying the reported experiences of the first COVID-19–related lockdown is likely to reflect differences in emotional burden, individual circumstances, and ways of coping with the pandemic, which is in line with previous research on this matter.

**Conclusions:**

This study illustrates the timely and efficient applicability of an NLP pipeline and thereby serves as a precedent for applied researchers. Our study thereby contributes to both the dissemination of NLP techniques in applied health sciences and the identification of previously unknown experiences and burdens of persons with MS during the pandemic, which may be relevant for future treatment.

## Introduction

Recent innovations in natural language processing (NLP) techniques and software have resulted in the emergence of numerous conveniently accessible and open-source analytical tools for the efficient evaluation of free-text data [1-4]. Textual data constitute a rich source of information, allowing the capture of unique perspectives, experiences, and individual needs through a broad range of different sources of information (eg, health-related content and emotional tone) [5,6]. While larger positive emotion vocabulary is linked to more mental well-being and better physical health, larger negative emotion vocabulary is associated with distress and decreased physical health [7].

In health research, the increasing availability of “real-world data” in the form of, for example, written text, constitutes a promising avenue to gain valid insights into themes that concern persons with chronic diseases in everyday life and thus are key to tailor individual support [8-10]. Many studies rely on interview techniques to gain such insights [11-16]. While conducting interviews represents the “gold standard” for gaining qualitative insights into individual experiences and perspectives, they may not always be feasible to assess individuals on a large scale. Scalable methods, which are very well suited for standardized quantitative assessments, may instead miss important information because they consist of predetermined items. Surveys that include open-ended text assessments can therefore be an appropriate way to qualitatively explore individual-level experiences and perspectives on a large scale in real-world environments.

Concurrently, practical guidelines for applied researchers concerning processing and evaluation procedures for textual information at a magnitude that is not feasible for manual analyses seem to be lacking. Given the novelty of the NLP method in the field of health research, we aim to share our work and experience in this manuscript to support applied researchers in implementing the NLP method in their own research. Therefore, the high-level aims of this study pertain to the investigation of the feasibility, usability, and scientific value of an NLP pipeline applied to the exploration of important life topics and themes in a large sample of persons with multiple sclerosis (MS) collected during a major health crisis. This study aims to provide practical guidance for applied researchers and leverages textual data from 639 well-documented persons with MS who described their live experiences during the first COVID-19 lockdown in Switzerland, as well as the availability of easy-to-use open-source tools for NLP.

At the content level, we addressed several specific research questions. We aimed to (1) identify cluster groups of persons with MS based on reported COVID-19–related topics; (2) determine the emotional tone underlying participants’ text entries; and (3) describe persons allocated to the same cluster group. For validation purposes, our analysis results were complemented by including independently collected information from the same database and a critical review by experts from the clinical or epidemiological research field.

## Methods

### Setting and Context

As laboratory-confirmed SARS-CoV-2 infections increased to up to almost 1500 cases daily (population size: 8.6 million inhabitants), the Swiss government implemented an initial lockdown between March 16 and April 27, 2020, to flatten the infection curve. On April 27, 2020, hairdressers, garden centers, flower shops, building supplies stores, and massage and beauty salons could reopen. In addition, entry requirements had been relaxed. On May 11, 2020, shops, restaurants, markets, libraries, and primary and secondary schools were reopened. The relaxations were accompanied by protection concepts. At the beginning of June 2020, all tourist facilities could open in compliance with protection measures. Events with up to 300 people could be held again, and gatherings with a maximum of 30 people were allowed again. On June 15, 2020, Switzerland lifted the entry regulations concerning all European Union/European Free Trade Association states and the United Kingdom. On June 19, 2020, the Swiss Federal Council lifted the state of emergency. Most COVID-19 measures were lifted from June 22, 2020 (exception: large events with over 1000 people remained forbidden until the end of August 2020). All places open to the public needed to have a protection concept [17,18]. This first lockdown in Switzerland due to the COVID-19 pandemic resulted in pervasive and high levels of distress and isolation in the general population. These repercussions had a disproportionate effect on vulnerable subgroups of the population already burdened with pre-existing chronic diseases, such as MS. During the early stages of the pandemic, MS was also considered a risk factor for more severe COVID-19 symptoms, and persons with MS were advised to strictly adhere to preventive measures (ie, staying at home and keeping physical distance). At the end of April 2020, the lockdown measures were gradually lifted.

### Data Sources

**Figure 1:**
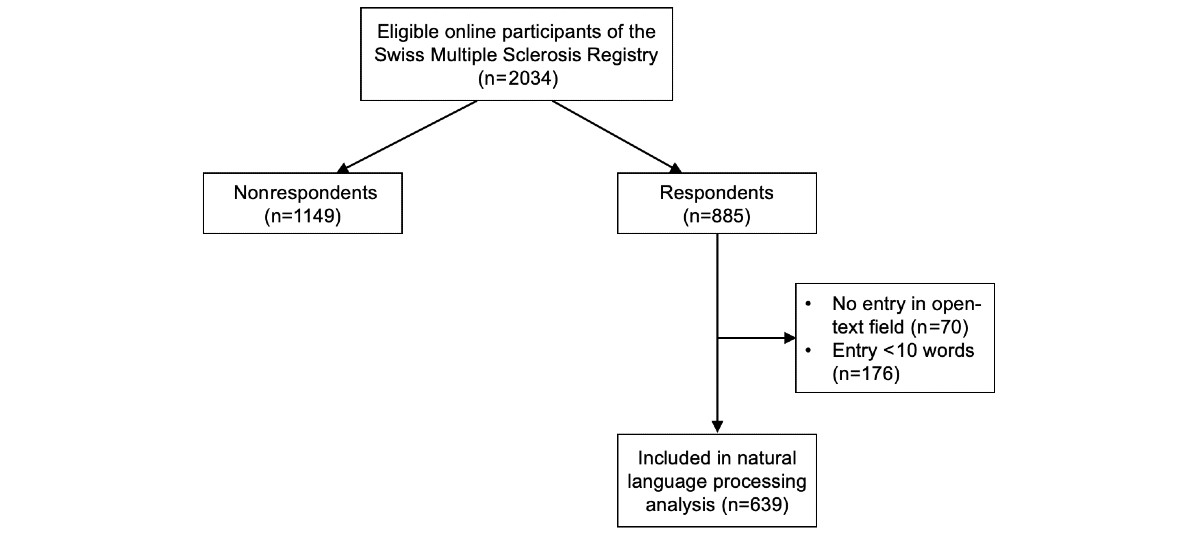
Flow diagram displaying the assessment procedure and subsequent selection procedure for online participants. Only online participants who described the experienced impact of COVID-19 on their personal life with at least 10 words were included in the text analysis.

To assess the impact of the lockdown on the everyday lives of persons with MS, the Swiss MS Registry conducted a COVID-19–focused online survey among its over 2500 participants (Figure 1). The Swiss MS Registry is a nationwide survey-based registry encompassing adults with MS who reside in or receive MS-related care in Switzerland.

The “COVID-19 survey” was a brief online survey released by the Swiss MS Registry in response to the lockdown measures for the first wave, which assessed mental well-being and difficulties in accessing health care in times of COVID-19. The complete survey is provided in Multimedia Appendix 1. The COVID-19 survey starts with a short introduction, followed by a section on mental well-being, in which depressive symptoms are assessed using the Beck Depression Inventory FastScreen questionnaire [19]. This is followed by an assessment of physical well-being (ie, possible worsening of health or MS symptoms), fear of the presence of a serious illness (eg, coronavirus) in addition to MS, and perceived loneliness. The survey finally assesses general changes in individuals’ life situations due to the coronavirus. The open question, which is analyzed in the present, concerned the pandemic’s perceived impact on respondents’ daily lives. Specifically, participants were asked the following question: “How does the current coronavirus situation affect your personal life (eg, in terms of social contacts, everyday tasks, and health care provision)?” Participants were invited to document their answers without a maximum word limit in either German, French, or Italian (ie, the 3 official languages of the Swiss MS Registry). The COVID-19 survey was released online on April 10, 2020, and remained accessible until October 31, 2020. The current analysis includes all data collected until September 7, 2020.

For this study, the COVID-19 survey data were combined with sociodemographic and health-related data collected as part of the semiannual Swiss MS Registry assessments preceding the COVID-19 survey. Specifically, we employed the Self-Reported Disability Status Scale (SRDSS) to determine MS physical gait impairments. In this regard, the SRDSS classifies gait impairments based on 2 self-report questions that assess walking distance and the use of assistance devices [20]. Further, we determined health-related quality of life using the EuroQol 5-dimension scale (EQ-5D; index and visual analog scale) [21].

### Ethics Approval

Approval has been obtained from the Cantonal Ethics Committee Zurich (PB-2016-00894). All participants enrolled in the Swiss MS Registry provided written (paper-pencil participants) or electronic (online participants) informed consent [22,23].

### Descriptive Statistics

To characterize and compare online participants from the Swiss MS Registry participating in the COVID-19 survey with nonparticipants, sociodemographic and health characteristics were analyzed by means of N (%) for categorical data and medians (IQR) for continuous data. Descriptive statistics were based on the brief entry questionnaire, which is mandatory for all Swiss MS Registry participants and includes information on age, sex, MS type, diagnosis date, and any disease-modifying treatments.

### Preprocessing and Analysis Pipeline for Free-Text Entries

This research implemented and evaluated a preprocessing and analysis pipeline to characterize and cluster free-text entries. To this end, we applied this pipeline to free-text entries about the impact of COVID-19 on the everyday lives of persons with MS. The entries were collected as part of the Covid-19 survey. The text preprocessing and analysis pipeline to be examined in this research consists of the following 5 interlinked consecutive steps: (1) text preprocessing; (2) descriptive text analysis; (3) sentiment analysis; (4) topic modeling; and (5) results interpretation and validation. An overview of the tools used in each step of the NLP pipeline can be found in Multimedia Appendix 2.

#### Step 1: Text Preprocessing

As the first step of the preprocessing procedure, Italian and French texts were translated into German using “DeepL Pro” [24], a tool for automatic text translation. Initially, we specified a cutoff for the minimum number of words for a text entry to be considered in the subsequent pipeline. As there are no generally valid guidelines applicable for our research in this regard, we based our decision on prior screening of the text entries and determined 10 words as cutoff to ensure sufficient informative content for the research question that we were interested in. Translation accuracy was checked manually and found to be very high. Further, punctuations and stop words (ie, common words without specific meaning like “the”) were removed using a publicly available German stop word list [25]. The remaining words were lemmatized (ie, changed to their root such as “studies” to “study”). Words not listed in dictionaries were converted into generic terms (eg, “Skype” to “video call”). This part of the pipeline was implemented using the Python library “spaCy” (version 2.3.2) [26].

#### Step 2: Descriptive Text Analyses

The second step of the pipeline concerned descriptive text analyses that involved determination of word frequencies as well as their visualization. For word frequency visualization, “word clouds” were compiled, which position all words into a graph where their relative size is determined by their overall frequency (ie, more frequent words are displayed larger in the plot) using the Python library “Wordcloud” (version 1.7.0) [27].

#### Step 3: Sentiment Analysis

The next step in pipeline pertained to the determination of linguistic indicators of overall text emotionality through sentiment analysis. To this end, 2 different text analysis resources were used: the well-established text analysis software “Linguistic Inquiry and Word Count” (LIWC) and further “SentimentWortschatz” (short “SentiWS”), a publicly available German-language resource for sentiment analysis. Sentiment analysis implemented in LIWC involved determining the text entries’ overall “emotional tone.” [28] “Emotional tone” is a summary variable provided by LIWC and represents the overall emotional coloration of a text. Scores range from 0 (negative tone) to 100 (positive tone), where a score of 50 indicates an even balance between positive and negative emotion words. Furthermore, we quantified text-based emotionality through “polarity scores” using the SentimentWortschatz sentiment-analysis resource (“SentiWS”) [29]. Polarity scores computed by SentiWS assess whether a word has a positive or negative connotation, ranging between −1 and 1. They are computed through a dictionary-based scoring algorithm that identifies words reflecting a negative or positive emotion. The SentiWS dictionary does not contain any polarity “shifters” or “intensifiers,” that is, words with an amplifying function, which weaken, intensify, or even reverse the meaning of an emotional word (eg, “not happy” or “very happy”). Since such amplifying words are key to accurately determine the polarity of a sentence, a German-language extension dictionary was used.

#### Step 4: Unsupervised Machine Learning–Topic Modeling

The final step of the pipeline concerns the implementation of “topic modeling,” which is an unsupervised text classification method with the aim to identify distinct clusters of common topics underlying free text (ie, underlying participants’ text entries) [30]. To determine distinct topic clusters, we implemented nonnegative matrix factorization, which is a topic modeling approach based on dimension reduction. Such dimension-reduction models are based on understanding a text corpus as a compilation of term frequencies. Nonnegative matrix factorization is based on a “bag of words” model, where text elements are represented in an unordered fashion. We further worked with unigrams, which means that each word corresponds to a text element (contrary to, for example, a bigram where a text element consists of 2 consecutive words). The reason for this methodological decision is that the majority of the words in the present data are meaningful in themselves in terms of co-occurrence and frequency.

We implemented this step using the Python libraries “scikit-learn” and “gensim” [31,32]. To determine the most suitable solution in terms of the number of distinct topics, we used the commonly used coherence score “C_v” as a criterion. “C_v” ranges from 0 (no topic coherence) to 1 (complete topic coherence). “C_v” scores for a modeling solution with 1 to 30 distinct topics are presented in Multimedia Appendix 3. We also computed the coherence score “UMass” but based the final topic modeling solution on “C_v” as it has been shown to be more appropriate for text data consisting of few words [33]. For sensitivity purposes, we repeated our analysis based on all available entries (ie, without word count restriction) in order to verify that topic clusters were stable.

#### Step 5: Results Interpretation and Validation

Finally, we labeled each of the distinct topic clusters with the term that occurred most often within the specific topic cluster. To further characterize individuals allocated to the distinct topic clusters, we compared independently collected sociodemographic measures across the groups through descriptive analyses. Given the descriptive nature of this research, we present 95% CIs instead of *P* values. We further linked emotional tone to the SRDSS score and years since diagnosis, which were both assessed as part of the previous biannual registry surveys. We also calculated the associations between emotional tone and the occurrence of new symptoms, the worsening of old symptoms, the presence of depressive symptoms, and the feeling of loneliness. For associations between interval-scaled variables, we calculated the Pearson correlation coefficient. For associations with ordinal variables, we computed the Spearman correlation coefficient. For correlations between interval-scaled and binary variables, we calculated the biserial point correlation coefficient. All associations were computed using the R package “psych” [34]. CIs for the Spearman correlation coefficient were computed using the R package “DescTools” [35]. Finally, the findings were critically reviewed by a team of experts coauthoring this study. The experts’ backgrounds and specialist knowledge include neurology, neuropsychology, and epidemiology, as well as a personal health history of MS.

## Results

### Sample Characteristics

**Figure 2:**
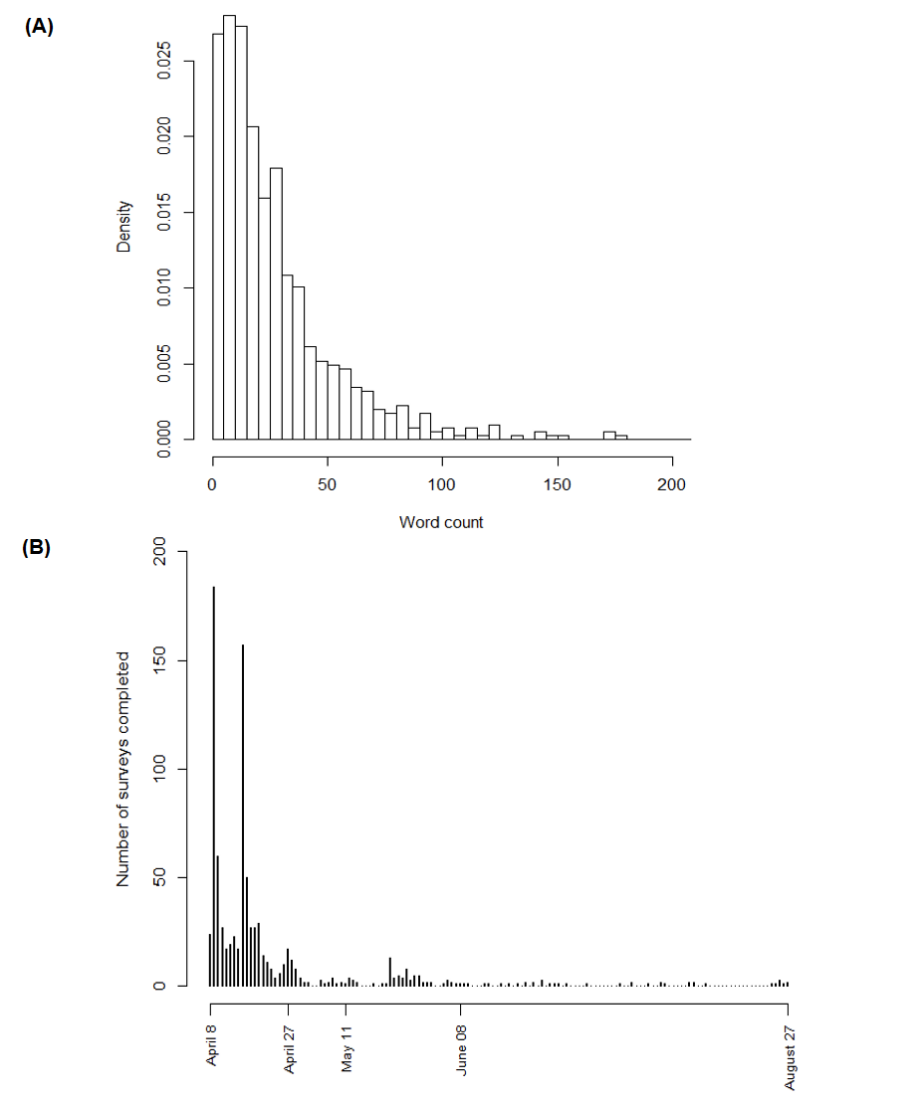
Survey responses included in this study. (A) Histogram depicting the text entries of different word lengths on the self-reported daily-life impact of COVID-19 (n=885). The number of words per text entry are plotted along the y-axis. (B) Amount of completed surveys across time (April 8, 2020, to August 27, 2020). Overall, 86.9% (555/639) of the responses were collected during the first lockdown (ie, before April 27, 2020). The number of completed surveys is displayed on the y-axis. Time (ie, days) is plotted along the x-axis.

A total of 885 Swiss MS Registry participants (44.5% of all participants) completed a questionnaire pertaining to COVID-19 (Figure 1). As presented in Table 1, COVID-19–related survey respondents had a median age of 48 years, 70.3% (622/885) were female, and 67.9% (601/885) had relapsing-remitting MS (that is, with intermittent recovery of acute MS symptoms as opposed to continuously worsening primary and secondary progressing MS). Overall, participants who completed the COVID-19 survey were similar to nonparticipants (n=1149) in terms of their baseline characteristics (median age 47 years, 72.6% [834/1149] female, and 66.9% [769/1149] relapsing-remitting MS). From the overall sample of available survey responses (n=885; study flow chart provided in Figure 1), this study focused on entries of at least 10 words (n=639; Figure 2A). As there are no generally valid guidelines applicable for our research in this regard, we based our decision on prior screening of the text entries and determined 10 words as cutoff to ensure sufficient informative content for the research question that we were interested in. From this data source, 639 entries were used for the text analyses in this study.

The following sections describe the results obtained from the text preprocessing and analysis pipeline, which was applied to a sample of 639 COVID-19–related text entries provided by the Swiss MS Registry participants. The rationale for the methodological decisions of this study is provided in the Methods section.

**Table 1:** Description of Swiss Multiple Sclerosis Registry online participants and nonparticipants.

Characteristic^a^		Nonparticipants (did not complete the COVID-19 survey; N=1149)	Participants (completed the COVID-19 survey; N=885)
**Age**			
	Value (years), median (IQR)	47.0 (38-56)	48.0 (39-56)
	Missing information, n (%)	50 (4.4)	25 (2.8)
**Gender, n (%)**			
	Female	834 (72.6)	622 (70.3)
	Male	315 (27.4)	262 (29.6)
	Missing information	0 (0)	1 (0.1)
**Language, n (%)**			
	German	903 (78.6)	695 (78.5)
	French	206 (17.9)	153 (17.3)
	Italian	40 (3.5)	37 (4.2)
**MS^b^ type, n (%)**			
	CIS^c^	31 (2.7)	16 (1.8)
	PPMS^d^	99 (8.6)	94 (10.6)
	RRMS^e^	769 (66.9)	601 (67.9)
	SPMS^f^	134 (11.7)	142 (16.0)
	Transition between 2 MS types or unspecified	30 (2.6)	27 (3.1)
	Missing information	86 (7.5)	5 (0.6)
**Disease-modifying MS medication (immunotherapy), n (%)**			
	Yes	285 (24.8)	586 (66.2)
	No	188 (16.4)	222 (25.1)
	Missing information	676 (58.8)	77 (8.7)
**Disease duration**			
	Value (years), median (IQR)	10.0 (5-18)	10.0 (4-17)
	Missing information, n (%)	104 (9.1)	34 (3.8)
**VAS^g^ (health-related QLS^h^)**			
	Value, median (IQR)	77 (54-90)	80 (60-90)
	Missing information	185 (16.1)	121 (13.7)
**EQ-5D^i^**			
	Value, median (IQR)	68.3 (49-88)	69.1 (51-91)
	Missing information	185 (16.1)	121 (13.7)

^a^Percentages were rounded and may thus not add up to 100%.

^b^MS: multiple sclerosis.

^c^CIS: clinically isolated syndrome.

^d^PPMS: primary progressive MS.

^e^RRMS: relapsing-remitting MS.

^f^SPMS: secondary progressive MS.

^g^VAS: visual analog scale.

^h^QLS: quality of life scale.

^i^EQ-5D: EuroQol 5-dimension scale.

### Descriptive Text Analyses

**Figure 3:**
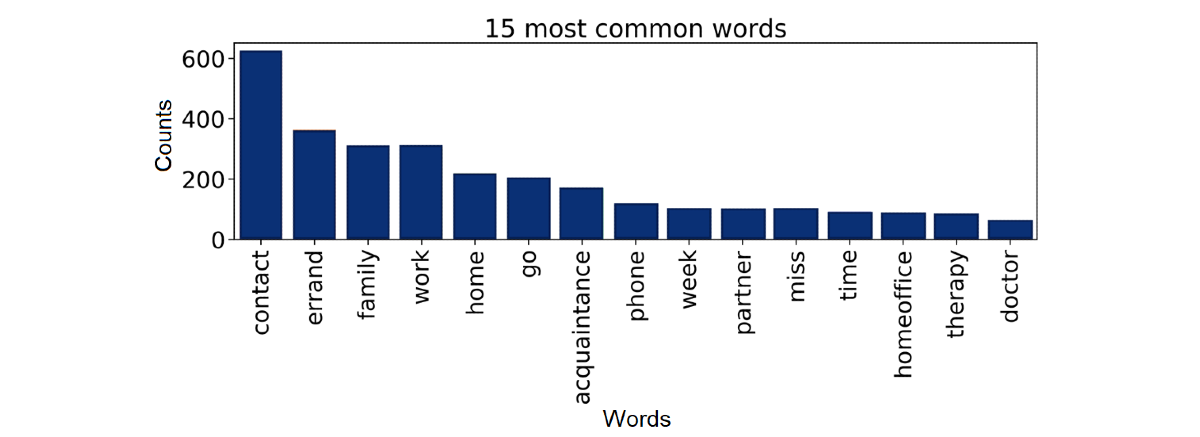
Most frequent keywords across free-text descriptions on participants’ perceived impact of COVID-19 on their personal life. Only text entries with at least 10 words in total were considered (n=639). “Stop words” (eg, “and” and “the”) were removed prior to the analysis.

Among all text responses used in this study, 86.9% (555/639) were collected during the first lockdown (before April 27, 2020; Figure 2B). In total, 80.1% (512/639) of these text entries were in German, 16.0% (102/639) in French, and 3.9% (25/639) in Italian. The median number of words per entry was 26 (IQR 16-44; following translation to German if necessary). Figure 3 visualizes the 15 most frequent keywords across the sample of text entries examined in this research. The most frequent words were “contact” (n=621), “errand” (n=364), “family” (n=307), “work” (n=307), and “home” (n=220).

### Sentiment Analysis

The possible full range of emotional tone of text entries ranged from 0 (negative) to 50 (neutral) up to 100 (positive). The mean emotional tone of participants’ text entries was 34.7 (SD 37.7), thus reflecting an overall negative emotional tone. The distribution of emotional tone quartiles (1st quartile: 0-24; 2nd quartile: 25-49; 3rd quartile: 50-74; 4th quartile: 75-100) revealed that most of the 639 entries fell into the 1st quartile and thus were of overall negative quality (439/639, 68.7%). Importantly, most of the remaining text entries fell into the 4th quartile and thus were unambiguously of positive quality (160/639, 25.0%), while only few text entries were allocated to the intermediate quartiles (2nd quartile: 7/639, 1.1%; 3rd quartile: 33/639, 5.2%). The skewed distribution of the emotional tone of the participants’ text entries explains the large standard deviation.

In terms of changes in COVID-19 measures across time, the average emotional tone across text entries did not differ during the lockdown (April 6 to 27; n=555; mean 35.32, SD 37.98; 95% CI 32.16-38.48) compared to the period during which restrictive measures were gradually lifted (April 28 to September 07; n=84; mean 30.58, SD 35.68; 95% CI 22.95-38.21).

Text-based polarity scores (ranging from −1 to 1) were comparable to those for emotional tone. Polarity scores were of overall negative valence (mean −0.10, SD 0.65), and 38.8% (248/639) of the entries had a polarity score below 0. Polarity scores based on text entries collected during first lockdown did not differ from those based on text entries collected during the time when measures were eased (following the lockdown; mean −0.13, SD 0.62).

### Unsupervised Learning–Topic Modeling

**Figure 4:**
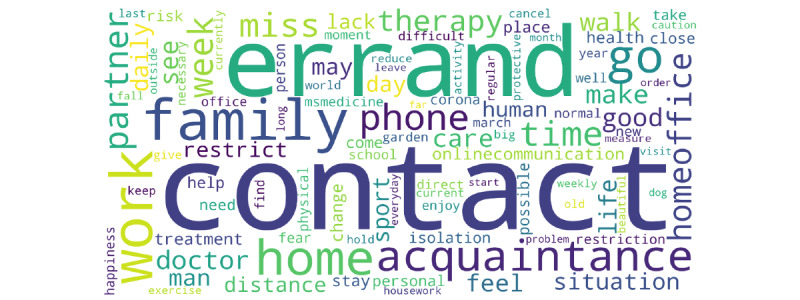
Word cloud visualizing the most frequent keywords related to the impact of COVID-19 on participants’ personal lives across the complete study sample. Word size reflects the relative frequency of a specific word in comparison to the total number of analyzed words. Only text entries with at least 10 words in total were considered (n=639).

Finally, the 639 text entries were grouped into distinct clusters through an unsupervised topic modeling procedure. Results revealed that a 4-group solution would be most suitable for the data structure. A word cloud visualizing the most frequent keywords related to the impact of COVID-19 on participants’ personal lives across the complete study sample can be found in Figure 4. Word clouds for the 4 distinct topic groups are provided in Multimedia Appendix 4. The 4 distinct “topic groups” were labeled with the most frequent keywords (group 1: “contacts/communication,” group 2: “social environment,” group 3: “work,” and group 4: “errands/daily routines”). A table characterizing the 4 distinct “topic groups” is provided in Multimedia Appendix 5. Text entries that were allocated to the “contacts/communication” group (group 1; 14.6% [119/639] of all text entries) captured how persons with MS experienced the contact restrictions. One of the most frequent words in this topic group was “miss.” Importantly, text entries allocated to this group were of increasingly negative polarity. On the other hand, polarity scores in the “social environment” group (group 2; 21.4% [174/639] of all entries) and “work” group (group 3; 17.9% [146/639] of all entries) were more balanced. Finally, the “errands/daily routines” group (group 4; 24.5% [200/639] of all entries) included keywords that reflected daily routines (eg, “errands” and “going for a walk”). This group included the largest percentage of positive polarity scores (56.5%, 113/200). Repetition of the topic modeling analyses using all available text entries consistently found modeling 4 topic clusters to be ideal.

### Sociodemographic and Health Characteristic Profiles

Additionally, we examined whether different sociodemographic and health characteristics were linked to distinct topic groups. The “contacts and communication” topic group tended to be older (median age: 49.5 years), live alone (27.7%, 33/119), be employed (second most; 63.9%, 76/119), and have lower levels of ambulatory disability (ie, persons who can move around without walking aids as measured with the self-reported disability scale [SRDSS], scores ranging between 0 and 3.5; 76.5%, 91/119). This group also reported the second highest health-related quality of life (median visual analog scale score: 80). Individuals allocated to the “social environment” topic group were more likely to have children (highest percentage; 50.6%, 88/174) who were typically under 18 years old (27.0%, 47/174). Further, pronounced mobility restrictions (ie, SRDSS scores greater than 3.5, thus requiring walking aids such as crutches or a wheelchair) were more frequent in this group, while health-related quality of life was comparatively lower (median EQ-5D: 0.65; median visual analog scale score: 75). Individuals allocated to the “work” topic group were most often employed compared to individuals in the other 3 topic groups (87.0%, 127/146), had SRDSS scores in the 0-3.5 range (highest proportion; 82.2%, 120/146), and had overall good quality of life (median EQ-5D index: 0.75; median visual analog scale score: 81). The “errands/daily routines” topic group had the most number of female research volunteers (79.0%, 158/200) and the highest proportion of persons on disability benefits (36.5%, 73/200). Quality of life in this group was higher as indicated by the visual analog scale (median score: 81). Finally, we examined the characteristics of online participants whose text entries had to be excluded as they were too short (n=176 entries). Individuals whose text entries had to be excluded were comparable to those of topic group 2 in terms of their sociodemographic characteristics (data not shown). Notably, the 3 most frequent keywords in the excluded entries (ie, “contacts,” n=64; “errands,” n=13; and “work,” n=10) were also present in the 4 topic groups.

We further examined whether emotional tone was linked to measures of physical or mental well-being. Emotional tone was not linked to the SRDSS score (rho=−0.02, 95% CI −0.09 to 0.06; S=39575496, *P*=.69) or the number of years since the initial MS diagnosis (*r*=−0.03, 95% CI −0.11 to 0.05; t_628_=−0.68333; *P*=.49). It was also not linked to the occurrence of new symptoms (*r*=−0.04, 95% CI −0.12 to 0.03; t_633_=−1.121; *P*=.26) or the worsening of new symptoms (*r*=−0.07, 95% CI −0.14 to 0.01; t_636_=−1.67; *P*=.09). However, emotional tone was significantly correlated with the presence of depressive symptoms (*r*=−0.10, 95% CI −0.19 to −0.02; t_627_=−2.49; *P*=.01) and feelings of loneliness (*r*=−0.12, 95% CI −0.18 to −0.02; t_630_=−2.92; *P*=.004). For all measures, less than 4% of the values were missing.

## Discussion

### Principal Findings

Here, we illustrate the application and subsequent evaluation of an NLP pipeline for the analysis of free-text data. Specifically, we applied this pipeline to text data on the experienced impact of the first COVID-19 lockdown from the perspectives of persons with MS collected by the Swiss MS Registry. Our study thus sheds light on individual daily-life experiences of the first COVID-19 lockdown in a vulnerable population.

In this study, we demonstrated both the feasibility and scientific value of an automated text preprocessing and NLP analysis pipeline based on existing open-source software in Python suitable for large-scale text data. The pipeline allows to preprocess real-world text data in an efficient fashion and to conduct timely and innovative analyses, including unsupervised machine learning. In light of a dearth of practical guidance for such real-world text data preprocessing and analysis procedures suitable for applied researchers, this pipeline has the potential to contribute to the dissemination of methodological knowledge, allowing to tap the potential of free-text data to capture individual perspectives and needs in health research. This study is embedded into the Swiss MS Registry, which is a large-scale well-documented longitudinal study. The registry’s data thus constitute an optimal use case for the application and evaluation of such a pipeline and the broad range of available data sources allowed that characterize individuals allocated to the distinct topic cluster groups in terms of specific characteristics. This study demonstrates the potential of open-ended questions in complementing traditional standardized assessment methods to capture unexplored information from individuals’ own words and thereby may spark new hypotheses and future avenues in health research. This type of language processing would essentially constitute a synergy between structured data collection and other forms of qualitative assessments, which tend to be more time-consuming in terms of processing and analysis (eg, interviews). Real-world data are afflicted with a broad range of challenges (eg, typos and dialect), which need elaborate consideration through text preprocessing to ensure the validity of subsequent complex analyses. Our study is thus timely and innovative in nature given its focus on key challenges when leveraging text data sources originating from a real-world setting through an efficient pipeline programmed in Python.

In terms of individual experiences of the first COVID-19 lockdown, the themes that concerned persons with MS most during the first COVID-19 lockdown differed substantially across study participants. Specifically, our study identified the following 4 distinct COVID-19–related topic groups, which participants could be assigned to based on their experiences: “contacts/communication” (group 1); “social environment” (group 2); “work” (group 3); and “errands/daily routines” (group 4). It is important to mention that between-group comparisons of sociodemographic and health-related characteristics corroborate the disparity of the 4 topic groups. This new topic-based approach to characterize persons with MS provides a novel perspective on individual experiences of the first COVID-19 lockdown and further highlights heterogeneity in terms of individual needs. To the best of our knowledge, there are no comparable in-depth studies researching the individually perceived impact of COVID-19 using participants own words. With regard to the overall emotional tone underlying the text entries, our findings revealed that most text entries reflected negative emotional states. This adds to research emphasizing the high burden of COVID-19–related restrictions for persons with MS given their prior vulnerability [12]. Further, from a methodological perspective, the context of our study was ideal for the identification of distinct topic commonalities of wide-ranging relevance as the spectrum of topics that participants were concerned with was confined. On the contrary, studies researching mundane everyday life situations of persons with MS are likely to identify considerably more diverse topics (with smaller population sizes per topic group), which results in the necessity of more data and participants, as suggested by an ongoing analysis of health diary entries collected before the COVID-19 pandemic from the same study population (manuscript in preparation).

In parallel with this finding, the 4 topic groups also differed in terms of the emotional tone underlying their text descriptions. It is important to mention that the emotional tone was determined through an independent analysis approach (sentiment analysis). A correlation analysis revealed that emotional tone was not associated with MS traits or measures of physical well-being, but with psychological well-being in the form of depressive symptoms and feelings of loneliness. This result suggests that “emotional tone” in this study primarily reflects emotions that are directly related to the content of the text and the individual’s situation. The most negative entries occurred in topic groups whose text entries predominately pertained to contacts and communication themes (group 1). In the topic groups concerning social environment (group 2) and work (group 3), the underlying emotional tone was more balanced, while in the topic group pertaining to errands and daily routines (group 4), the entries’ emotional tone was predominantly positive. This observed heterogeneity in emotional tonality underlying the reported experiences of the first COVID-19–related lockdown is likely to reflect differences in emotional burden, individual circumstances, and ways of coping with the pandemic, which is in line with previous research in this matter. For instance, a US telephone survey on persons with MS conducted during the first lockdown found that a higher perceived impact of the pandemic on individuals’ self-reported psychological well-being was linked to a higher impact of MS symptoms on individuals’ daily lives. Further, by conducting interviews, a recent study found that persons reporting no or even a positive impact of the pandemic on their lives tended to cope with the pandemic situation with active problem-focused strategies [11-13]. In terms of personal values, however, another study examining young persons with MS also reported perceived positive effects of the pandemic situation in the form of personal, relational, and existential growth [36]. Accordingly, participants allocated to the “contacts and communication” topic group made the highest number of negative text entries and reported the lowest quality of life (median). Taken together, these findings are foreground to the burdensome effects of the pandemic in terms of isolation, and reduction or even loss of social contact/activities and personal exchange in vulnerable individuals such as persons with MS. Based on the sociodemographic and disease characteristics of topic group 1, feelings of isolation appeared exacerbated in persons with MS who were comparatively less impaired or living alone. This finding might be related to the fact that persons with high disease burden are more accustomed to daily life restrictions compared to those with less impairments.

### Limitations

Despite its notable strengths, the present research has some limitations, which merit consideration. First, there is a dearth of well-established guidelines for NLP that consider the specificities of health research. Consequently, the implementation of different text classification modeling approaches might have resulted in slightly divergent clusters and overarching topics. As such, to examine the robustness of our findings, we reanalyzed our data using the well-established Latent Dirichlet Allocation approach, which yielded similar patterns compared to those reported (not shown in this article) and thus corroborates the robustness of the presented results. Topic modeling further groups frequently co-occurring words into clusters (ie, “topics”). This method is suitable for identifying topics underlying large-scale text data in a data-driven fashion to thereby generate novel insights that might have been missed by standardized quantitative assessments. Our study does, however, not provide information to specifically tailor MS treatment to the needs of an individual person. The emotional tone indicates a general trend of the overall valence of a topic, while there may be variations at the individual level. Our findings have revealed experiences and burdens of persons with MS during the COVID-19 pandemic that may be relevant to future treatments or may provide insights for future research. Further limitations pertain to the generalizability of the findings of the sample population to the total population of persons with MS in Switzerland. Participants of this study constitute a subsample of the Swiss MS Registry’s participants. The registry itself covers the diversity of the Swiss population of persons with MS in terms of a broad range of characteristics [37]. The participants of the MS Registry subsample who completed the “COVID-19 survey” were comparatively younger, less disabled, and residing more often in the German-speaking region of Switzerland than the nonparticipants of the registry. However, we did not find any indications for systematic differences between the linguistic regions. The translation of non-German text entries into German through an automated translation software is afflicted with the risk of potential mistranslations, misinterpretations, and biases. However, it is important to mention that both exploratory count comparison of the most frequent keywords and manual spot-checking were not suggestive of any systematic differences across languages.

### Conclusion

We demonstrated the potential of a preprocessing and NLP analysis pipeline for large-scale text data and applied it to COVID-19–related data collected by the Swiss MS Registry, which constitutes an optimal use case for the pipeline. Above and beyond providing practical guidance for applied researchers, our study has implications for efficiently leveraging large-scale textual data in health care settings. Electronic health records and clinical notes have received increasing attention as rich sources of information, which are accessible through the application of NLP techniques [38-40].

Our study further demonstrates an approach that complements structured and standardized assessments through individual participant perspectives and hence provides ecologically valid information. We provide practical guidance for applied health researchers who wish to follow a similar approach by (1) demonstrating the processing and analysis process using large-scale real-world data and (2) providing a detailed description of the pipeline, which is based (apart from LIWC) on freely available open-source software. Interested researchers can follow both the entire process and the software we use. Given the novelty of the emerging NLP field, we are, in this way, contributing to the establishment of good practice standards and the dissemination of knowledge around NLP methodology among applied researchers, especially those from the health sciences.

## Appendix

Multimedia Appendix 1 COVID-19 survey of the Swiss Multiple Sclerosis Registry.

Multimedia Appendix 2 An overview of the tools used in each step of the natural language processing pipeline.

Multimedia Appendix 3 Graph showing topic coherence scores (blue dots) for topic models on the experience of the first COVID-19 lockdown in persons with multiple sclerosis, with 1 to 30 distinct topics. The number of modeled topics is plotted along the x-axis. Coherence scores are plotted along the y-axis. Topic coherence refers to the semantic similarity of words allocated to a distinct topic and constitutes a key goodness of fit measure for topic models. The full possible range of coherence scores is between 0 (no topic coherence) and 1 (complete topic coherence). A 4-topic model provides the optimal modeling solution for the data as indicated by the highest coherence score.

Multimedia Appendix 4 Word clouds visualizing the most frequent keywords related to the impact of COVID-19 on volunteers’ personal lives presented separately for each of the 4 topic cluster groups. Word size reflects the relative frequency of a specific word in comparison to the total number of analyzed words in a topic group. Only text entries with at least 10 words in total were considered.

Multimedia Appendix 5 Characterization of study participants assigned to the 4 topic groups “contacts/communication,” “social environment,” “work,” and “errands/daily routines”.

## Abbreviations

EQ-5D: EuroQol 5-dimension scale
LIWC: Linguistic Inquiry and Word Count
MS: multiple sclerosis
NLP: natural language processing
SentiWS: SentimentWortschatz
SRDSS: Self-Reported Disability Status Scale

## References

[1] Cammel SA, De Vos MS, van Soest D, Hettne KM, Boer F, Steyerberg EW, Boosman H (2020). How to automatically turn patient experience free-text responses into actionable insights: a natural language programming (NLP) approach. BMC Med Inform Decis Mak.

[2] Dreisbach C, Koleck TA, Bourne PE, Bakken S (2019). A systematic review of natural language processing and text mining of symptoms from electronic patient-authored text data. Int J Med Inform.

[3] Koleck TA, Dreisbach C, Bourne PE, Bakken S (2019). Natural language processing of symptoms documented in free-text narratives of electronic health records: a systematic review. J Am Med Inform Assoc.

[4] Mascio A, Kraljevic Z, Bean D, Dobson R, Stewart R, Bendayan R, Roberts A (2005). Comparative Analysis of Text Classification Approaches in Electronic Health Records. arXiv.

[5] Calvo RA, Milne DN, Hussain MS, Christensen H (2017). Natural language processing in mental health applications using non-clinical texts. Nat. Lang. Eng.

[6] Elkin PL, Mullin S, Mardekian J, Crowner C, Sakilay S, Sinha S, Brady G, Wright M, Nolen K, Trainer J, Koppel R, Schlegel D, Kaushik S, Zhao J, Song B, Anand E (2021). Using Artificial Intelligence With Natural Language Processing to Combine Electronic Health Record's Structured and Free Text Data to Identify Nonvalvular Atrial Fibrillation to Decrease Strokes and Death: Evaluation and Case-Control Study. J Med Internet Res.

[7] Vine V, Boyd RL, Pennebaker JW (2020). Natural emotion vocabularies as windows on distress and well-being. Nat Commun.

[8] Rivas R, Montazeri N, Le NX, Hristidis V (2018). Automatic Classification of Online Doctor Reviews: Evaluation of Text Classifier Algorithms. J Med Internet Res.

[9] Ferrario A, Demiray B, Yordanova K, Luo M, Martin M (2020). Social Reminiscence in Older Adults' Everyday Conversations: Automated Detection Using Natural Language Processing and Machine Learning. J Med Internet Res.

[10] Le Glaz A, Haralambous Y, Kim-Dufor D, Lenca P, Billot R, Ryan TC, Marsh J, DeVylder J, Walter M, Berrouiguet S, Lemey C (2021). Machine Learning and Natural Language Processing in Mental Health: Systematic Review. J Med Internet Res.

[11] Donisi V, Gajofatto A, Mazzi MA, Gobbin F, Busch IM, Ghellere A, Rimondini M (2020). Insights for Fostering Resilience in Young Adults With Multiple Sclerosis in the Aftermath of the COVID-19 Emergency: An Italian Survey. Front Psychiatry.

[12] Morris-Bankole H, Ho AK (2021). The COVID-19 Pandemic Experience in Multiple Sclerosis: The Good, the Bad and the Neutral. Neurol Ther.

[13] Talaat F, Ramadan I, Aly S, Hamdy E (2020). Are multiple sclerosis patients and their caregivers more anxious and more committed to following the basic preventive measures during the COVID-19 pandemic?. Mult Scler Relat Disord.

[14] Vogel AC, Schmidt H, Loud S, McBurney R, Mateen FJ (2020). Impact of the COVID-19 pandemic on the health care of >1,000 People living with multiple sclerosis: A cross-sectional study. Mult Scler Relat Disord.

[15] Manacorda T, Bandiera P, Terzuoli F, Ponzio M, Brichetto G, Zaratin P, Bezzini D, Battaglia MA (2021). Impact of the COVID-19 pandemic on persons with multiple sclerosis: Early findings from a survey on disruptions in care and self-reported outcomes. J Health Serv Res Policy.

[16] Colais P, Cascini S, Balducci M, Agabiti N, Davoli M, Fusco D, Calandrini E, Bargagli AM (2021). Impact of the COVID-19 pandemic on access to healthcare services amongst patients with multiple sclerosis in the Lazio region, Italy. Eur J Neurol.

[17] (2020). Easing and tightening of nationwide measures. Federal Office of Public Health.

[18] (2022). Coronavirus: Measures and ordinances. Federal Office of Public Health.

[19] Kliem S, Mößle T, Zenger M, Brähler E (2014). Reliability and validity of the Beck Depression Inventory-Fast Screen for medical patients in the general German population. J Affect Disord.

[20] Kaufmann M, Salmen A, Barin L, Puhan MA, Calabrese P, Kamm CP, Gobbi C, Kuhle J, Manjaly Z, Ajdacic-Gross V, Schafroth S, Bottignole B, Ammann S, Zecca C, D'Souza M, von Wyl V, Swiss Multiple Sclerosis Registry (SMSR) (2020). Development and validation of the self-reported disability status scale (SRDSS) to estimate EDSS-categories. Mult Scler Relat Disord.

[21] Hinz A, Klaiberg A, Brähler E, König HH (2006). [The Quality of Life Questionnaire EQ-5D: modelling and norm values for the general population]. Psychother Psychosom Med Psychol.

[22] Puhan MA, Steinemann N, Kamm CP, Müller S, Kuhle J, Kurmann R, Calabrese P, Kesselring J, von Wyl V, Swiss Multiple Sclerosis Registry (SMSR) (2018). A digitally facilitated citizen-science driven approach accelerates participant recruitment and increases study population diversity. Swiss Med Wkly.

[23] Steinemann N, Kuhle J, Calabrese P, Kesselring J, Disanto G, Merkler D, Pot C, Ajdacic-Gross V, Rodgers S, Puhan MA, von Wyl V, Swiss Multiple Sclerosis Registry (SMSR) (2018). The Swiss Multiple Sclerosis Registry (SMSR): study protocol of a participatory, nationwide registry to promote epidemiological and patient-centered MS research. BMC Neurol.

[24] DeepL Translator. DeepL.

[25] (2020). Stopwords ISO. Gene Diaz.

[26] (2022). Industrial-strength natural language processing in Python. spaCy.

[27] Oesper L, Merico D, Isserlin R, Bader GD (2011). WordCloud: a Cytoscape plugin to create a visual semantic summary of networks. Source Code Biol Med.

[28] Meier T, Boyd RL, Pennebaker JW, Mehl MR, Martin M, Wolf M, Horn AB “LIWC auf Deutsch”: The Development, Psychometrics, and Introduction of DE- LIWC2015. PsyArXiv Preprints.

[29] Remus R, Quasthoff U, Heyer G (2010). SentiWS - A Publicly Available German-language Resource for Sentiment Analysis. Proceedings of the Seventh International Conference on Language Resources and Evaluation (LREC'10).

[30] Blei D, Carin L, Dunson D (2010). Probabilistic Topic Models: A focus on graphical model design and applications to document and image analysis. IEEE Signal Process Mag.

[31] Pedregosa F, Varoquaux G, Gramfort A, Michel V, Thirion B, Grisel O, Blondel M, Prettenhofer P, Weiss R, Dubourg V (2011). Scikit-learn: Machine Learning in Python. The Journal of Machine Learning Research.

[32] Rehurek R, Sojka P (2010). Software Framework for Topic Modelling with Large Corpora. Proceedings of the LREC 2010 Workshop on New Challenges for NLP Frameworks.

[33] Röder M, Both A, Hinneburg A (2015). Exploring the Space of Topic Coherence Measures. WSDM '15: Proceedings of the Eighth ACM International Conference on Web Search and Data Mining.

[34] Revelle W (2022). psych: Procedures for Psychological, Psychometric, and Personality Research. R Project homepage.

[35] Signorell A, Aho K, Alfons A, Anderegg N, Aragon T, Arachchige C, Arppe A (2022). DescTools: Tools for Descriptive Statistics. R Project.

[36] Poli S, Rimondini M, Gajofatto A, Mazzi MA, Busch IM, Gobbin F, Schena F, Del Piccolo L, Donisi V (2021). "If You Can't Control the Wind, Adjust Your Sail": Tips for Post-Pandemic Benefit Finding from Young Adults Living with Multiple Sclerosis. A Qualitative Study. Int J Environ Res Public Health.

[37] Kaufmann M, Puhan MA, Kuhle J, Yaldizli Ö, Magnusson T, Kamm CP, Calabrese P, von Wyl V (2019). A Framework for Estimating the Burden of Chronic Diseases: Design and Application in the Context of Multiple Sclerosis. Front Neurol.

[38] Sheikhalishahi S, Miotto R, Dudley JT, Lavelli A, Rinaldi F, Osmani V (2019). Natural Language Processing of Clinical Notes on Chronic Diseases: Systematic Review. JMIR Med Inform.

[39] Yang X, Zhang H, He X, Bian J, Wu Y (2020). Extracting Family History of Patients From Clinical Narratives: Exploring an End-to-End Solution With Deep Learning Models. JMIR Med Inform.

[40] Spasic I, Nenadic G (2020). Clinical Text Data in Machine Learning: Systematic Review. JMIR Med Inform.

