# Supplementary material for: The Real-World Experiences of Persons With Multiple Sclerosis During the First COVID-19 Lockdown: Application of Natural Language Processing
Source: JMIR Med Inform. 2022 Nov 10;10(11):e37945. doi: 10.2196/37945 (PMC9651007; doi:10.2196/37945)
Supplement: Multimedia Appendix 1 [file medinform_v10i11e37945_app1.pdf]

Additional survey for current reasons (coronavirus)

In the light of the current situation, we at the MS Registry, together with our colleagues from the Department of Neurology and Psychiatry at the University of Bern, would like to find out what consequences the current wave of coronavirus disease has on your personal wellbeing.

The coronavirus disease wave concerns us all. For you as a person with MS, the current measures and recommendations to stay away from public life may also have drastic consequences. We now want to record this burden, which can worsen both the physical and the psychological condition, in order to better understand the effects for persons with MS.

We would therefore like to invite you to participate in this additional survey. With your information, we can compare the current data with the entries from previous surveys. We hope that this deeper understanding will help us to better treat persons with MS in such an uncertain situation in the future. We would like to take this opportunity to thank you for your help.

The topic blocks of this additional survey cover the following main areas:

- Questions about your psychological wellbeing with a focus on depressiveness and worry.
- Questions about your physical wellbeing with a focus on MS relapses and recurrence of old symptoms.

This additional questionnaire will usually take **no more than 15 minutes** to complete. Please fill out the questionnaire completely even if everything has remained the same for you in the past months or you do not feel particularly affected by the current situation. This information is also very valuable for recording the situation of persons with MS in Switzerland.

Start questionnaire

Part A: Mental wellbeing

BDI FastScreen *(7-item self-report questionnaire for the detection of depression; for further information please see: [21])*

Part B: Physical wellbeing

Below you will find some questions about your physical well-being.

B1 Have you had any new symptoms of MS recently (since February 2020)?

Please select only one of the following answers.

- Yes
- No

If yes, which ones?

(If applicable:)

B2 Have you seen a doctor about these symptoms?

Please select only one of the following answers.

- Yes
- No

If yes, what kind of appointment

- Personal visit to doctor
- Telephone consultation with doctor

If you did not consult a doctor, please state the reason:

B3 If you have seen a doctor, these complaints have been assessed by the doctor as a relapse?

Please select only one of the following answers.

- Yes
- No

B4 If yes, was the episode treated and with what?

Please select only one of the following answers.

- Yes
- No

Treated with:

B5 Have you had any worsening of old, known symptoms recently (since February 2020)?

Please select only one of the following answers.

- Yes
- No

If yes, which:

B6 On a scale from 1 (not at all) to 5 (very pronounced)....

	**1** (not at all)	**2**	**3**	**4**	**5** (very pronounced)
... how much do you currently suffer from a fear of having a serious illness (such as coronavirus) in addition to MS, without it already having been diagnosed by a doctor?	○	○	○	○	○
... how lonely do you feel at the moment?	○	○	○	○	○

Part C General changes due to the coronavirus

Below you will find a question about the general changes in your situation caused by the coronavirus.

C1 How does the current Coronavirus situation affect your personal life (e.g., in terms of social contacts, everyday tasks, health care provision)?

End of the questionnaire

This was the last question in the current supplementary survey on the topic of coronavirus. In the current stressful situation, we would like to point out once again where you can get help if you need it.

If you are looking for advice or a helpful, supportive conversation, you can contact the MS-Infoline of the Swiss MS Society. MS Society (0844 674 636).

The Swiss helpline can be reached around the clock by calling 143 (‘Die Dargebotene Hand’). This service is also a suitable first contact in particularly difficult and urgent situations. It guarantees absolute anonymity and can help you find the right contacts for further counselling or treatment (for example, psychiatric emergency service or crisis intervention center in your region).

Once again, we would like to thank you very much for your important commitment and the time you take to fill in the questionnaires!
