# Supplementary figures and images for: The Real-World Experiences of Persons With Multiple Sclerosis During the First COVID-19 Lockdown: Application of Natural Language Processing
Source: JMIR Med Inform. 2022 Nov 10;10(11):e37945. doi: 10.2196/37945 (PMC9651007; doi:10.2196/37945)

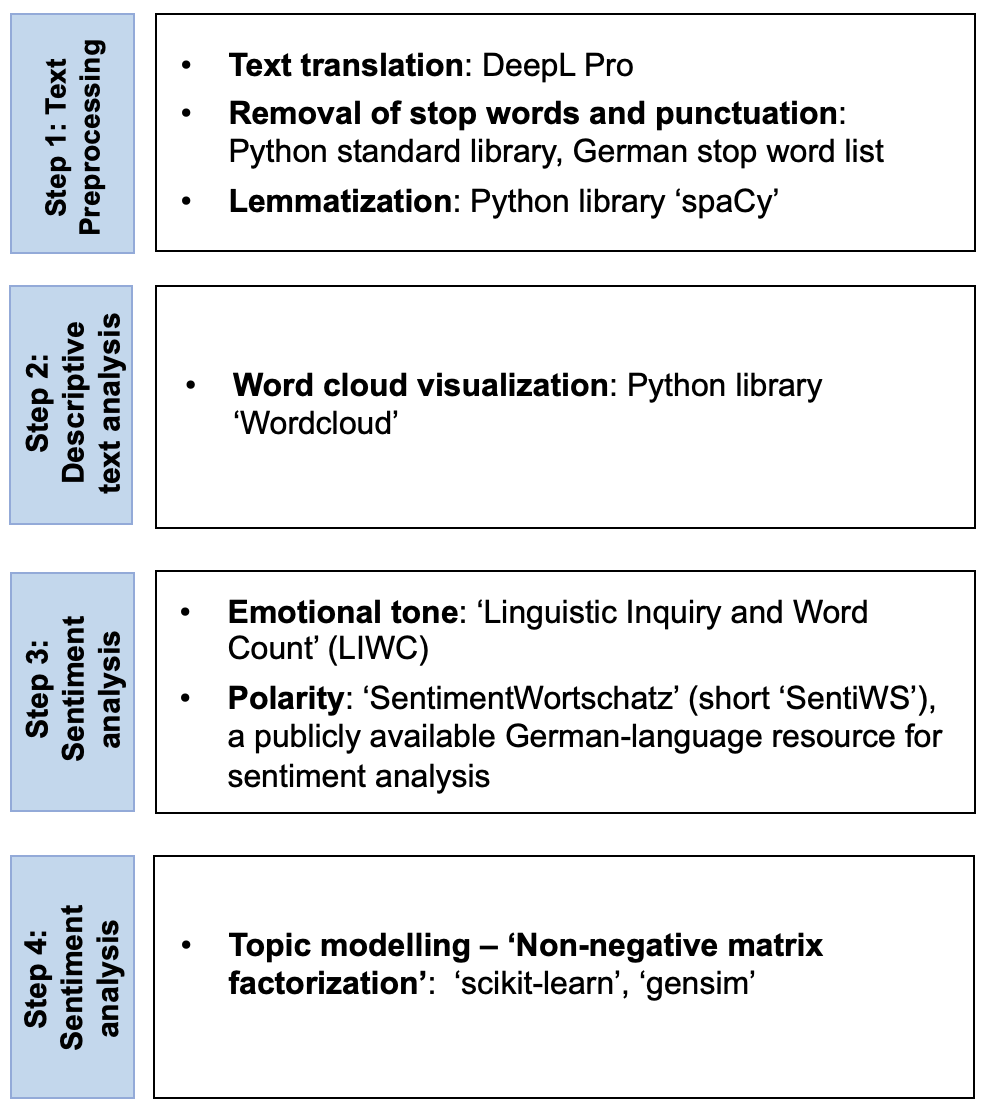

Supplement: Multimedia Appendix 2 [file medinform_v10i11e37945_app2.png]

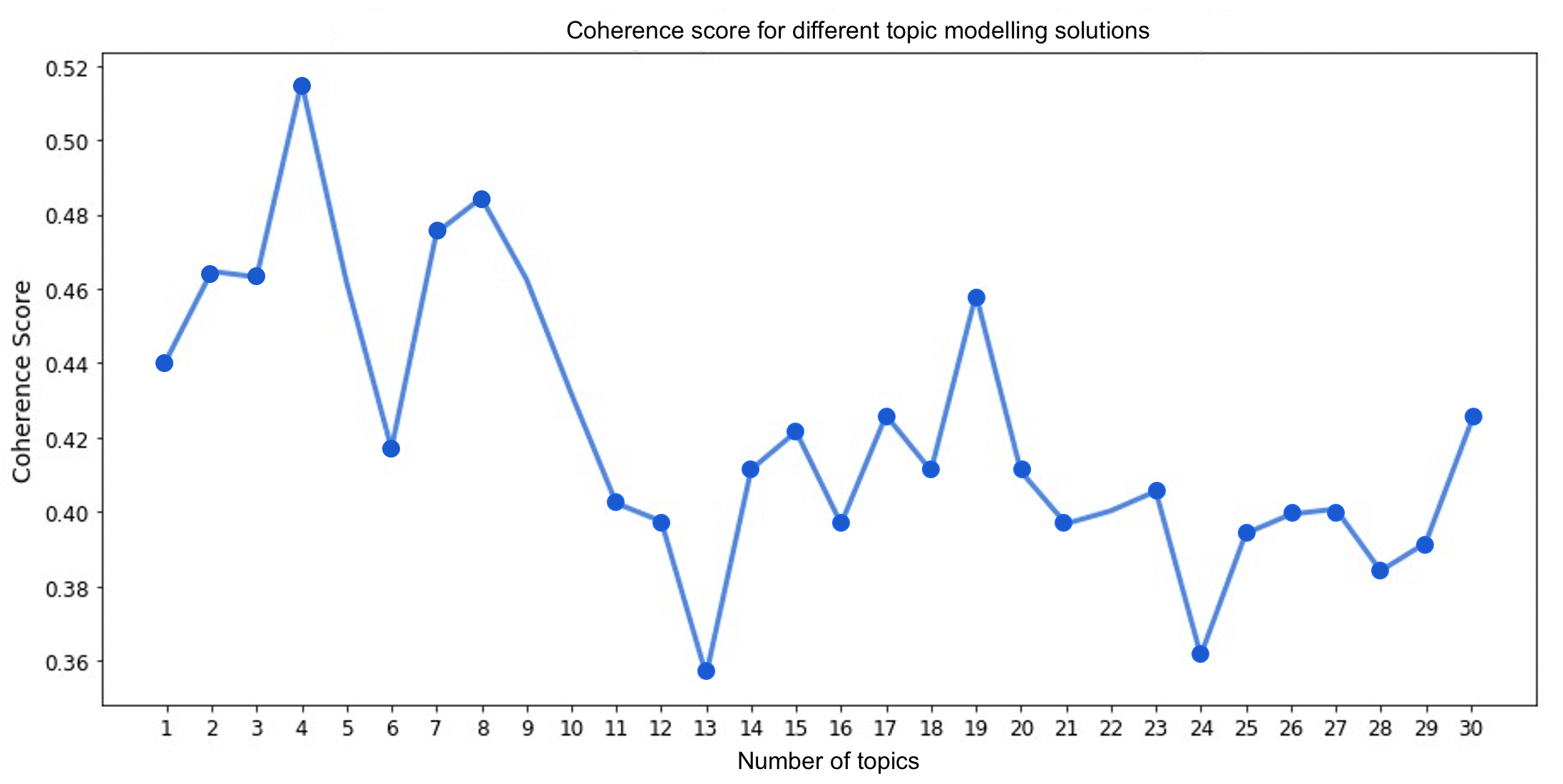

Supplement: Multimedia Appendix 3 [file medinform_v10i11e37945_app3.png]

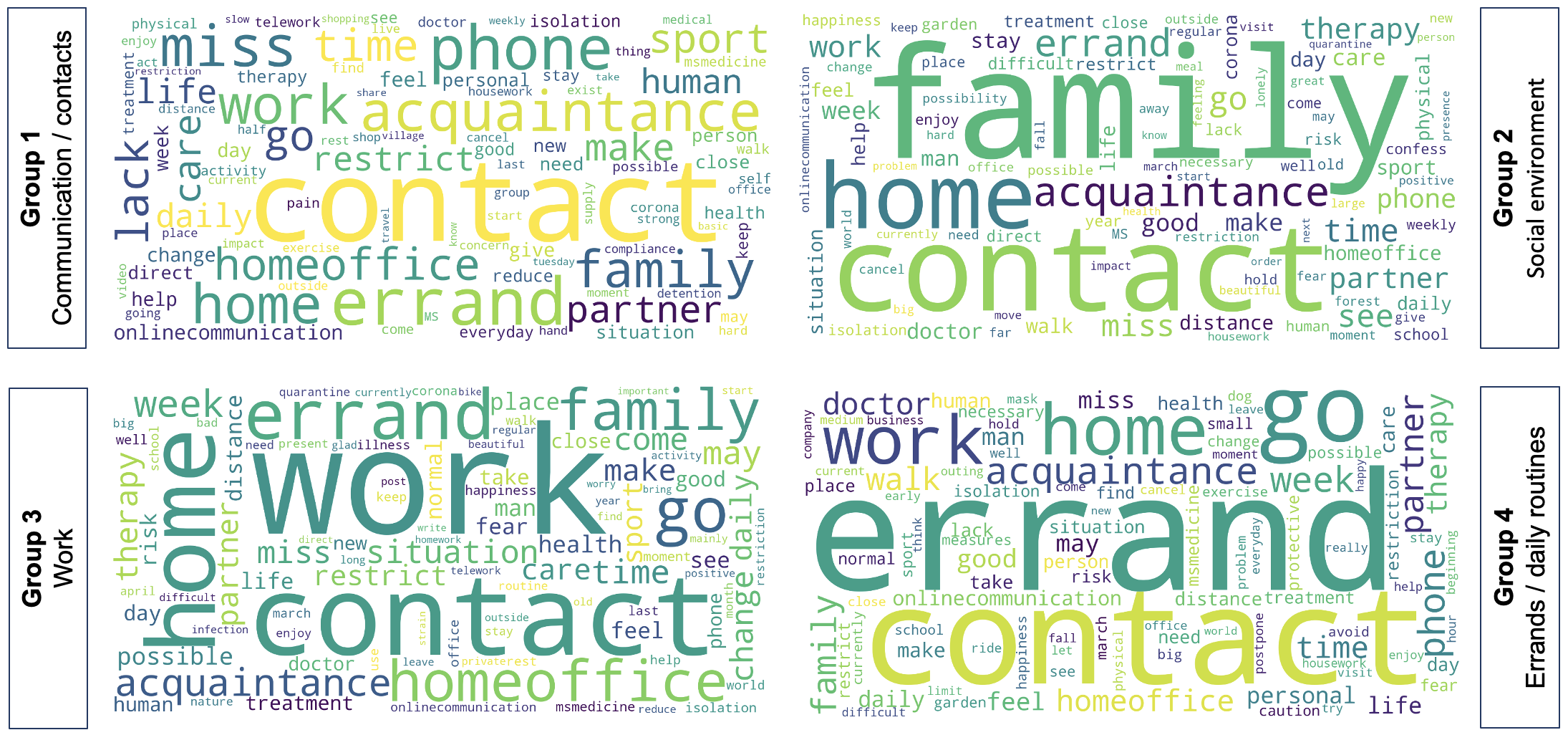

Supplement: Multimedia Appendix 4 [file medinform_v10i11e37945_app4.png]
