# Supplementary material for: The Real-World Experiences of Persons With Multiple Sclerosis During the First COVID-19 Lockdown: Application of Natural Language Processing
Source: JMIR Med Inform. 2022 Nov 10;10(11):e37945. doi: 10.2196/37945 (PMC9651007; doi:10.2196/37945)
Supplement: Multimedia Appendix 5 [file medinform_v10i11e37945_app5.docx]

|  | **Topic group** | | | |
| --- | --- | --- | --- | --- |
|  | **Contacts / Communication**  (n=119, 14.6%) | **Social environment**  (n = 174, 21.4%) | **Work**  (n = 146, 17.9%) | **Errands /**  **daily routines**  (n = 200, 24.5%) |
| **Keywords** | *• contact*  *• miss*  *• telephone*  *• acquaintances*  *• sports* | *• family*  *• friends*  *• contact*  *• home* | *• work*  *• at home*  *• home-office*  *• contact* | *• errands*  *• go*  *• week*  *• partner* |
| **Word count**  Median (IQR) | 23 [14-34] | 28 [17-51.75] | 26 [16 - 40] | 26 [16 - 44.25] |
| **Polarity score**  Mean (SD) | -0.16 | -0.08 | -0.11 | -0.07 |
| Median (IQR) | 0 [-0.34 - 0.004] | 0 [-0.38 - 0.22] | 0 [-0.33 - 0.11] | 0.004 [-0.30 - 0.22] |
| Proportion of text entries with positive polarity score | 0.328 | 0.425 | 0.401 | 0.565 |
| Proportion of text entries with negative polarity scores | 0.437 | 0.402 | 0.38 | 0.35 |
|  |  |  |  |  |
| **Age (years)** |  |  |  |  |
| median(IQR) | 49.5 [40 - 56] | 48 [39 - 58.5] | 47 [36 - 53] | 48 [41 - 58] |
| mean(SD) | 48.1 (11.8) | 49.2 (12.6) | 45.2 (11.1) | 49.5 (11.8) |
| 95% CI for mean | [45.87 - 50.23] | [47.34 - 51.14] | [43.33 - 47.02] | [47.87 - 51.22] |
| **Sex**  [95%-CI for proportion] |  |  |  |  |
| Female | 84 (70.6%) | 128 (73.6%) | 107 (73.3%) | 158 (79%) |
|  | [0.61 - 0.78] | [0.66 - 0.80] | [0.65 - 0.80] | [0.73 - 0.84] |
| Male | 35 (29.4%) | 46 (26.4%) | 39 (26.5%) | 42 (21%) |
|  | [0.22, 0.39] | [0.20, 0.34] | [0.20, 0.35] | [0.16, 0.27] |
| **Language**  [95%-CI for proportion] |  |  |  |  |
| German | 89 (74.8%) | 130 (74.7%) | 113 (76.9%) | 180 (90%) |
|  | [0.66 - 0.82] | [0.67 - 0.81] | [0.70 - 0.84] | [0.85 - 0.94] |
| French | 25 (21%) | 34 (19.5%) | 27 (18.5%) | 16 (8%) |
|  | [0.14 - 0.30] | [0.14 - 0.26] | [0.13 - 0.26] | [0.05 - 0.13] |
| Italian | 5 (4.2%) | 10 (5.7%) | 6 (4.1%) | 4 (2%) |
|  | [0.02 - 0.10] | [0.03 - 0.11] | [0.02 - 0.09] | [0.01 - 0.05] |
| **Education**  [95%-CI for proportion] |  |  |  |  |
| Mandatory school completed | 1 (1%) | 2 (1.2%) | 2 (1.4%) | 0 (0%) |
|  | [0.00 - 0.05] | [0.00 - 0.05] | [0.00 - 0.05] | [0.00 - 0.02] |
| Highschool | 12 (10%) | 9 (5.2%) | 16 (11%) | 10 (5%) |
|  | [0.06 - 0.17] | [0.03 - 0.10] | [0.07 - 0.17] | [0.03 - 0.09] |
| Apprenticeship | 31 (26%) | 47 (27%) | 27 (18.5%) | 89 (44.5%) |
|  | [0.19 - 0.35] | [0.21 - 0.34] | [0.13 - 0.26] | [0.38 - 0.52] |
| Higher professional education | 19 (15.9%) | 27 (15.5%) | 16 (11%) | 26 (13%) |
|  | [0.10 - 0.24] | [0.11 - 0.22] | [0.07 - 0.17] | [0.09 - 0.19] |
| University | 32 (26.9%) | 45 (25.9%) | 44 (30.1%) | 39 (19.5%) |
|  | [0.19 - 0.36] | [0.20 - 0.33] | [0.23 - 0.38] | [0.14 - 0.26] |
| Others | 2 (1.7%) | 1 (0.5%) | 4 (2.7%) | 3 (1.5%) |
|  | [0.00 - 0.07] | [0.00 - 0.04] | [0.01 - 0.07] | [0.00 - 0.05] |
| Missing information | 22 (18.5%) | 43 (24.7%) | 37 (25.3%) | 33 (16.5%) |
| **MS type**  [95%-CI for proportion] |  |  |  |  |
| CIS | 2 (1.7%) | 3 (1.7%) | 3 (2.1%) | 1 (0.5%) |
|  | [0.00 - 0.07] | [0.00 - 0.05] | [0.01 - 0.06] | [0.00 - 0.03] |
| RRMS | 82 (68.9%) | 104 (59.8%) | 116 (79.5%) | 146 (73.0%) |
|  | [0.60 - 0.77] | [0.52 - 0.67] | [0.72 - 0.86] | [0.66 - 0.79] |
| PPMS | 16 (13.4%) | 18 (10.3%) | 8 (5.5%) | 22 (11.0%) |
|  | [0.08 - 0.21] | [0.06 - 0.16] | [0.03 - 0.11] | [0.07 - 0.16] |
| SPMS | 11 (9.2%) | 43 (24.7%) | 13 (8.9%) | 28 (14.0%) |
|  | [0.05 - 0.16] | [0.19 - 0.32] | [0.05 - 0.15] | [0.1 - 0.2] |
| Transition between two stages and other forms | 6 (5.0%) | 5 (2.9%) | 6 (4.1%) | 2 (1.0%) |
|  | [0.02 - 0.11] | [0.01 - 0.07] | [0.02 - 0.09] | [0 - 0.04] |
| Missing information | 2 (1.7%) | 1 (0.6%) | 0 (0%) | 1 (0.5%) |
| **Disease-modifying MS Medication (immunotherapy)**  [95%-CI for proportion] |  |  |  |  |
| Yes | 80 (67.2%) | 123 (70.7%) | 106 (72.6%) | 140 (70.0%) |
|  | [0.58 - 0.75] | [0.63 - 0.77] | [0.64 - 0.79] | [0.63 - 0.76] |
| No | 30 (25.2%) | 39 (22.4%) | 26 (17.8%) | 47 (23.5%) |
|  | [0.18 - 0.34] | [0.17 - 0.29] | [0.12 - 0.25] | [0.18 - 0.3] |
| Missing information | 9 (7.6%) | 12 (6.9%) | 14 (9.6%) | 13 (6.5%) |
| **Marital status** |  |  |  |  |
| Divorced | 12 (10.1%) | 10 (5.7%) | 11 (7.5%) | 19 (9.5%) |
|  | [0.06 - 0.17] | [0.03 - 0.11] | [0.04 - 0.13] | [0.06 - 0.15] |
| Separated | 0 (0%) | 1 (0.6%) | 1 (0.7%) | 3 (1.5%) |
|  | [0 - 0.04] | [0 - 0.04] | [0 - 0.04] | [0 - 0.05] |
| Single | 38 (31.9%) | 28 (16.1%) | 44 (30.1%) | 58 (29.0%) |
|  | [0.24 - 0.41] | [0.11 - 0.23] | [0.23 - 0.38] | [0.23 - 0.36] |
| Registered partnership | 0 (0%) | 0 (0%) | 1 (0.7%) | 2 (1.0%) |
|  | [0 - 0.04] | [0 - 0.03] | [0 - 0.04] | [0 - 0.04] |
| Married | 46 (38.7%) | 93 (53.4%) | 51 (34.9%) | 80 (40.0%) |
|  | [0.3 - 0.48] | [0.46 - 0.61] | [0.27 - 0.43] | [0.33 - 0.47] |
| Widowed | 1 (0.8%) | 2 (1.1%) | 0 (0%) | 5 (2.5%) |
|  | [0 - 0.05] | [0 - 0.05] | [0 - 0.03] | [0.01 - 0.06] |
| Other | 0 (0%) | 1 (0.6%) | 2 (1.4%) | 2 (1.0%) |
|  | [0 - 0.04] | [0 - 0.05] | [0 - 0.04] | [0 - 0.04] |
| Missing information | 22 (18.5%) | 39 (22.4%) | 36 (24.7%) | 31 (15.5%) |
| **Living situation**  [95%-CI for proportion] |  |  |  |  |
| Alone | 33 (27.7%) | 27 (15.5%) | 22 (15.1%) | 55 (27.5%) |
|  | [0.2 - 0.37] | [0.11 - 0.22] | [0.1 - 0.22] | [0.22 - 0.34] |
| At a clinic/nursing home | 0 (0%) | 0 (0%) | 0 (0%) | 1 (0.5%) |
|  | [0 - 0.04] | [0 - 0.03] | [0 - 0.03] | [0 - 0.03] |
| Others | 1 (0.8%) | 1 (0.6%) | 2 (1.4%) | 3 (1.5%) |
|  | [0 - 0.05] | [0 - 0.04] | [0 - 0.05] | [0 - 0.05] |
| With family | 23 (19.3%) | 68 (39.1%) | 44 (30.1%) | 46 (23.0%) |
|  | [0.13 - 0.28] | [0.32 - 0.47] | [0.23 - 0.38] | [0.17 - 0.3] |
| With friends | 2 (1.7%) | 1 (0.6%) | 5 (3.4%) | 4 (2.0%) |
|  | [0 - 0.07] | [0 - 0.04] | [0.01 - 0.08] | [0.01 - 0.05] |
| With parents | 5 (4.2%) | 4 (2.3%) | 5 (3.4%) | 2 (1.0%) |
|  | [0.02 - 0.1] | [0.01 - 0.06] | [0.01 - 0.08] | [0 - 0.04] |
| With partner | 46 (38.7%) | 66 (37.9%) | 62 (42.5%) | 80 (40.0%) |
|  | [0.3 - 0.48] | [0.31 - 0.46] | [0.34 - 0.51] | [0.33 - 0.47] |
| Missing information | 9 (7.6%) | 7 (4.0%) | 6 (4.1%) | 9 (4.5%) |
| **Has children (yes/no)**  [95%-CI for proportion] |  |  |  |  |
| Yes | 46 (38.7%) | 88 (50.6%) | 47 (32.2%) | 86 (43.0%) |
|  | [0.3 - 0.48] | [0.43 - 0.58] | [0.25 - 0.4] | [0.36 - 0.5] |
| No | 47 (39.5%) | 36 (20.7%) | 58 (39.7%) | 69 (34.5%) |
|  | [0.31 - 0.49] | [0.15 - 0.28] | [0.32 - 0.48] | [0.28 - 0.42] |
| **Has children <=18 years (based on birth year of children)**  [95%-CI for proportion] |  |  |  |  |
| Yes | 17 (14.3%) | 47 (27.0%) | 28 (19.2%) | 34 (17.0%) |
|  | [0.09 - 0.22] | [0.21 - 0.34] | [0.13 - 0.27] | [0.12 - 0.23] |
| No | 30 (25.2%) | 40 (23.0%) | 18 (12.3%) | 49 (24.5%) |
|  | [0.18 - 0.34] | [0.17 - 0.3] | [0.08 - 0.19] | [0.19 - 0.31] |
| **Number of Children** |  |  |  |  |
| Mean (SD) | 1.00 (1.14) | 1.40 (1.10) | 0.971 (1.24) | 1.10 (1.18) |
| **Currently (self-)employed?**  [95%-CI for proportion] |  |  |  |  |
| Yes | 76 (63.9%) | 90 (51.7%) | 127 (87.0%) | 117 (58.5%) |
|  | [0.54 - 0.72] | [0.44 - 0.59] | [0.8 - 0.92] | [0.51 - 0.65] |
| No | 36 (30.3%) | 80 (46.0%) | 17 (11.6%) | 78 (39.0%) |
|  | [0.22 - 0.39] | [0.38 - 0.54] | [0.07 - 0.18] | [0.32 - 0.46] |
| Missing information | 7 (5.9%) | 4 (2.3%) | 2 (1.4%) | 5 (2.5%) |
| **Work percentage** |  |  |  |  |
| Median (IQR) | 90 [50 - 100] | 55 [30 - 80] | 80 [50 - 100] | 60 [40 - 100] |
| Mean (SD) | 75.8 (28.0) | 59.5 (30.2) | 73.2 (26.0) | 66.5 (29.9) |
| 95% CI for mean | [69.70 - 81.91] | [53.46 - 65.46] | [68.69 - 77.78] | [61.06 - 71.87] |
| **Receiving disability benefits (yes/no)**  [95%-CI for proportion] |  |  |  |  |
| Yes | 30 (25.2%) | 52 (29.9%) | 28 (19.2%) | 73 (36.5%) |
|  | [0.18 - 0.34] | [0.23 - 0.37] | [0.13 - 0.27] | [0.3 - 0.44] |
| No | 74 (62.2%) | 101 (58.0%) | 107 (73.3%) | 108 (54.0%) |
|  | [0.53 - 0.71] | [0.5 - 0.65] | [0.65 - 0.8] | [0.47 - 0.61] |
| Requested | 7 (5.9%) | 16 (9.2%) | 7 (4.8%) | 14 (7.0%) |
|  | [0.03 - 0.12] | [0.06 - 0.15] | [0.02 - 0.1] | [0.04 - 0.12] |
| Missing information | 8 (6.7%) | 5 (2.9%) | 4 (2.7%) | 5 (2.5%) |
| **Disease duration (years)** |  |  |  |  |
| Median(IQR) | 8 [4 - 15] | 11 [6 - 19] | 7 [3 - 14] | 12 [6 - 19] |
| Mean(SD) | 10 (7.3) | 12.8 (8.7) | 9.7 (8.3) | 13.1 (8.7) |
| 95%-CI for mean | [8.65 - 11.35] | [11.45 - 14.08] | [8.27 - 11.09] | [11.83 - 14.29] |
| **SRDSS** [95%-CI for proportion] |  |  |  |  |
| EDSS 0-3.5 (no walking aids) | 91 (76.5%) | 108 (62.1%) | 120 (82.2%) | 147 (73.5%) |
|  | [0.68 - 0.84] | [0.54 - 0.69] | [0.75 - 0.88] | [0.67 - 0.79] |
| EDSS 4-6.5 (use of walking aids) | 17 (14.3%) | 39 (22.4%) | 15 (10.3%) | 34 (17.0%) |
|  | [0.09 - 0.22] | [0.17 - 0.29] | [0.06 - 0.17] | [0.12 - 0.23] |
| EDSS 7-10 (use of wheelchair) | 3 (2.5%) | 21 (12.1%) | 8 (5.5%) | 12 (6.0%) |
|  | [0.01 - 0.08] | [0.08 - 0.18] | [0.03 - 0.11] | [0.03 - 0.1] |
| Missing information | 8 (6.7%) | 6 (3.4%) | 3 (2.1%) | 7 (3.5%) |
|  |  |  |  |  |
| **Health care providers they regularly see**  [95%-CI for proportion] |  |  |  |  |
| Neurologist | 105 (88.2%) | 152 (87.3%) | 131 (89.7%) | 178 (89%) |
|  | [0.81 - 0.93] | [0.81 - 0.92] | [0.83 - 0.94] | [0.84 - 0.93] |
| General practitioner | 67 (56.3%) | 113 (65%) | 85 (58.2%) | 129 (64.5%) |
|  | [0.47 - 0.65] | [0.57 - 0.72] | [0.5 - 0.66] | [0.57 - 0.71] |
| Physiotherapy/ergotherapy | 45 (37.8%) | 81 (46.6%) | 51 (35%) | 91 (45.5%) |
|  | [0.29 - 0.47] | [0.39 - 0.54] | [0.27 - 0.43] | [0.39 - 0.53] |
| Psychologist/psychiatrist | 21 (17.6%) | 29 (16.6%) | 16 (11%) | 32 (16%) |
|  | [0.11 - 0.26] | [0.12 - 0.23] | [0.07 - 0.17] | [0.11 - 0.22] |
|  |  |  |  |  |
| **Health related quality of life (EQ-5D): sum score** |  |  |  |  |
| Median (IQR) | 76 [56 - 93] | 64.6 [43 - 84] | 75 [59 - 91] | 71 [53 - 91] |
| Mean (SD) | 70 (26) | 60 (33) | 70 (26) | 67 (28) |
| 95% CI for mean | [65 - 75] | [55 - 66] | [66 - 75] | [63 - 71] |
|  |  |  |  |  |
| **Health related quality of life (EQ-5D): VAS** |  |  |  |  |
| Median (IQR) | 80 [63.5 - 90] | 75 [60 - 90] | 81 [65.25 - 90] | 81 [67.75 - 90] |
| Mean (SD) | 73.7 (20.0) | 70.9 (23.0) | 75.8 (20.3) | 75.9 (20.8) |
| 95%-CI for mean | [69.83 - 77.53] | [67.26 - 74.58] | [72.14 - 79.54] | [72.83 - 78.91] |
| **Depressive symptoms (BDI-II)** |  |  |  |  |
| Mean (SD) | 2.16 [2.83] | 1.85 [2.43] | 1.75 [2.37] | 1.82 [2.60] |
| Median (IQR) | 1.00 [3.00] | 1.00 [3.00] | 1.00 [3.00] | 1.00 [2.00] |
| 95%-CI for mean |  |  |  |  |
|  |  |  |  |  |
| **Loneliness** |  |  |  |  |
| Mean (SD) | 2.03 (1.16) | 1.74 (0.983) | 1.78 (1.07) | 1.94 (1.10) |
| Median (IQR) | 2.00 [2.00] | 1.00 [1.00] | 1.00 [1.00] | 2.00 [1.00] |
| 95%-CI for mean |  |  |  |  |

*Abbreviations*. BDI-II = Beck Depression Inventory; CI = confidence interval; CIS = Clinically Isolated Syndrome; IQR = interquartile range; PPMS = Primary Progressive MS; Polarity score = Score quantifying text-based with values ranging between -1 and + 1. Negative values refer to negative emotionality, whereas positive values indicate overall positive emotionality. Percentages were rounded and thus may not add up to 100%; SD = standard deviation; missing = information not provided by a certain number of participants; MS = Multiple Sclerosis; RRMS = relapsing-remitting Multiple Sclerosis; SPMS = Secondary Progressive MS; SRDSS = Self-report Disability Status Scale; EDSS = Expanded Disability Status Scale; SPMS = secondary progressive Multiple Sclerosis; VAS = visual analogue scale.
